# The Role of TGF-β/SMAD Signaling in Hepatocellular Carcinoma: from Mechanism to Therapy and Prognosis

**DOI:** 10.7150/ijbs.89568

**Published:** 2024-02-04

**Authors:** Xin Xin, Xiyu Cheng, Fanxin Zeng, Qing Xu, Lingling Hou

**Affiliations:** 1 The College of Life Science and Bioengineering, Beijing Jiaotong University, Beijing, China.; 2 Department of Clinical Research Center, Dazhou Central Hospital, Dazhou, Sichuan province, China.

**Keywords:** Hepatocellular carcinoma, TGF-β signaling, SMAD, Mechanism, Therapy

## Abstract

Hepatocellular carcinoma (HCC) is one of the most common cancers worldwide, with high incidence and mortality, accounting for approximately 90% of liver cancer. The development of HCC is a complex process involving the abnormal activation or inactivation of multiple signaling pathways. Transforming growth factor-β (TGF-β)/Small mothers against decapentaplegic (SMAD) signaling pathway regulates the development of HCC. TGF-β activates intracellular SMADs protein through membrane receptors, resulting in a series of biological cascades. Accumulating studies have demonstrated that TGF-β/SMAD signaling plays multiple regulatory functions in HCC. However, there is still controversy about the role of TGF-β/SMAD in HCC. Because it involves different pathogenic factors, disease stages, and cell microenvironment, as well as upstream and downstream relationships with other signaling pathways. This review will summary the regulatory mechanism of the TGF-β/SMAD signaling pathway in HCC, involving the regulation of different pathogenic factors, different disease stages, different cell populations, microenvironments, and the interaction with microRNAs. In addition, we also introduced small molecule inhibitors, therapeutic vaccines, and traditional Chinese medicine extracts based on targeting the TGF-β/SMAD signaling pathway, which will provide future research direction for HCC therapy targeting the TGF-β/SMAD signaling pathway.

## 1. Introduction

Liver cancer is a commonly diagnosed type of cancer, where abnormal cells grow in the liver or intrahepatic bile ducts. The global burden of liver cancer is substantial. A projection of incidence and mortality indicates that more than one million people will be affected by liver cancer in 2025^1^. Hepatocellular carcinoma (HCC) is the most common type of liver cancer, accounting for approximately 90% of all cases, and is the fourth leading cause of cancer-related deaths worldwide^2^. According to the American Cancer Society Global Cancer Statistics 2022, the 5-year relative survival rate for liver cancer was 20% from the mid-1970s to 2017, making it the second most deadly tumor after pancreatic cancer^3^. In recent decades, although the incidence of liver cancer has declined in some Asian countries such as Korea, Japan, and China, it continues to show an increasing trend in the United States, Australia, and much of Europe. Of these, Mongolia has the highest incidence and mortality rate in the world^4^.

The physiological and pathological changes of liver cancer are affected by multiple factors. Chronic hepatitis C (CHC), hepatitis B (HBV), and alcoholic liver disease (ALD) are the main risk factors in the development of liver cancer^4^. In addition, epidemiological surveys in recent years have shown that metabolic syndrome (MS), diabetes mellitus type 2 (T2DM), obesity, and non-alcoholic fatty liver disease (NAFLD) are also increasing in HCC patients, and may become the main risk factors for HCC in western countries^5^. Statistics on the proportion of liver cancers caused by different factors indicate that about 56% of liver cancers are associated with HBV, 20% with HCV, and about 20% with alcohol and smoking^6^. Significantly, NAFLD-associated HCC are increasing worldwide. The number of cases will increase from 10,820 to 24,860 in the USA and from 2,200 to 3,240 in Japan between 2016 and 2030^7^. Therefore, whether HCC is caused by viral hepatitis, alcohol, or non-alcoholic factors, it is a health challenge that cannot be ignored.

TGF-β/SMAD signaling pathway is a highly conserved molecular mechanism that regulates hepatobiliary development and cell fate during embryogenesis, as well as liver homeostasis and repair in adulthood^8^. It affects many human diseases, such as fibrosis, cancers, and connective tissue diseases, by regulating cell proliferation, apoptosis, differentiation, motility, lineage specificity, and stem cell homeostasis^8^. Interestingly, the TGF-β/SMAD signaling pathway plays a dual role in tumor. In the early stages of tumor, it mainly induces cell apoptosis and inhibits cell proliferation. However, in the late stage, tumor progression is promoted through epithelial-mesenchymal transition (EMT), angiogenesis, and immune escape. Likewise, the functional role and molecular mechanism of TGF-β/SMAD signaling in liver diseases are determined by pathogenic factors, development stage, and cell microenvironment. In addition, TGF-β receptor or SMADs mutations and epigenetic changes are often found in cancer types such as colon, pancreatic, and stomach cancers. Remarkably, these genetic changes are relatively rare in HCC^9^. At present, studies have shown that the TGF-β/SMAD signaling pathway is associated with poor prognosis in HCC^10^, however, their regulatory mechanisms are more complicated. The current review focuses on the role and possible mechanisms of the TGF-β/SMAD signaling in HCC and how these mechanisms may serve as potential targets for treatment and prognosis.

## 2. TGF-β/SMAD Signaling Pathway

### 2.1 TGF-β Family

TGF-β was first isolated in a serum-free medium from embryonic fibroblasts transformed by mouse sarcoma virus in 1978. So far, five TGF-β subtypes (TGF-β 1-5) have been identified, TGF-β 1-3 exists in mammalian cells, and the amino acid homology between them is 70-82%^11^. TGF-β1 is a well-studied member of the TGF family and plays a major role in tissue fibrosis and tumor progression. It activates fibroblasts and promotes the deposition of extracellular matrix (ECM)^12^. Accumulating evidence have demonstrated that increased expression of TGF-β1 is associated with tumor aggressiveness and poor prognosis in HCC^13^, bladder cancer^14^, breast cancer^15^, prostate cancer^16^ and cholangiocarcinoma^17^. Besides that, it has been indicated that TGF-β1 deficiency leads to abnormal hematopoietic and endothelial cell differentiation and autoimmune diseases^18^. TGF-β2 was first discovered in human glioblastoma, also known as a glioblastoma-derived T cell suppressor factor (G-TsF)^19^. Studies have shown that the expression of TGF-β2 in the aqueous humor of cataract patients is higher than that of TGF-β1 and TGF-β3, suggesting that TGF-β2 may be involved in the fibrosis process of lens epithelial cells^20^. TGF-β3 is originally identified from a cDNA library of a human rhabdomyosarcoma cell line and shares 80% amino acid sequence homology with TGF-β1 and TGF-β2, which plays a role in regulating EMT and is essential for lung and palate development in mice^21^. TGF-β3 mutation is associated with aortic aneurysms, mezzanine, and mitral valve disease, and other cardiovascular diseases^22^. TGF-β4 and TGF-β5 are present in birds and amphibians respectively, and but their biological roles are not well understood. Karaffová et al. reported that TGF-β4 helped to increase IgA secretion in poultry intestines^23^. TGF-β5 has been found to be expressed in the posterior end of the embryonic notochord in the early embryo of the African clawed toad^24^.

### 2.2 SMADs Family

SMADs are the core proteins of the TGF-β signaling cascade. They were first identified in Drosophila melanogaster and as part of members of the Decapentaplegic (Dpp) signaling pathway^25^. Subsequently, Cathy et al. found that the mutant phenotypes of sma-3 and sma-4 were identical with the TGF-β-like receptor gene daf-4 during investigating the genetic screening of Caenorhabditis elegans (C. elegans). Therefore, researchers assume that the Sma gene in C. elegans and the Mad gene in Drosophila are homologous genes. The Sma and Mad genes have also been shown to be homologous between humans and mice, and encode a family of proteins collectively known as the SMADs family^26^, ^27^. So far, eight SMADs family members have been found in mammals^28^, among which SMAD1, SMAD5 and SMAD8 (also known as SMAD9) have activation effects, while SMAD6 and SMAD7 exert inhibitory effects^29^. The SMADs family proteins are divided into three subfamilies based on their structure and function: (1) Receptor-regulated SMADs (R-SMADs), including SMAD1, SMAD2, SMAD3, SMAD5, and SMAD8. R-SMADs are involved in the specificity of signaling pathways, SMAD2 and SMAD3 mediate the TGF-β signaling pathway; SMAD1, SMAD5, and SMAD8 mediate the bone morphogenic protein (BMP) signaling pathway. (2) Common SMAD (Co-SMAD), currently, only SMAD4 has been found, is a binding ligand for all R-SMADs and facilitates the translocation of R-SMADs to the nucleus, which in turn regulates the transcription of target genes. (3) Inhibitory SMADs (I-SMAD), including SMAD6 and SMAD7, are negative regulators of the TGF-β/SMAD signaling pathway, competitively binding to TβRI to prevent phosphorylation of R-SMADs or directly inhibiting the transcriptional activity of SMAD multimers, further blocking the biological effects of the TGF-β signaling pathway^28^, ^30^.

**Figure 1:**
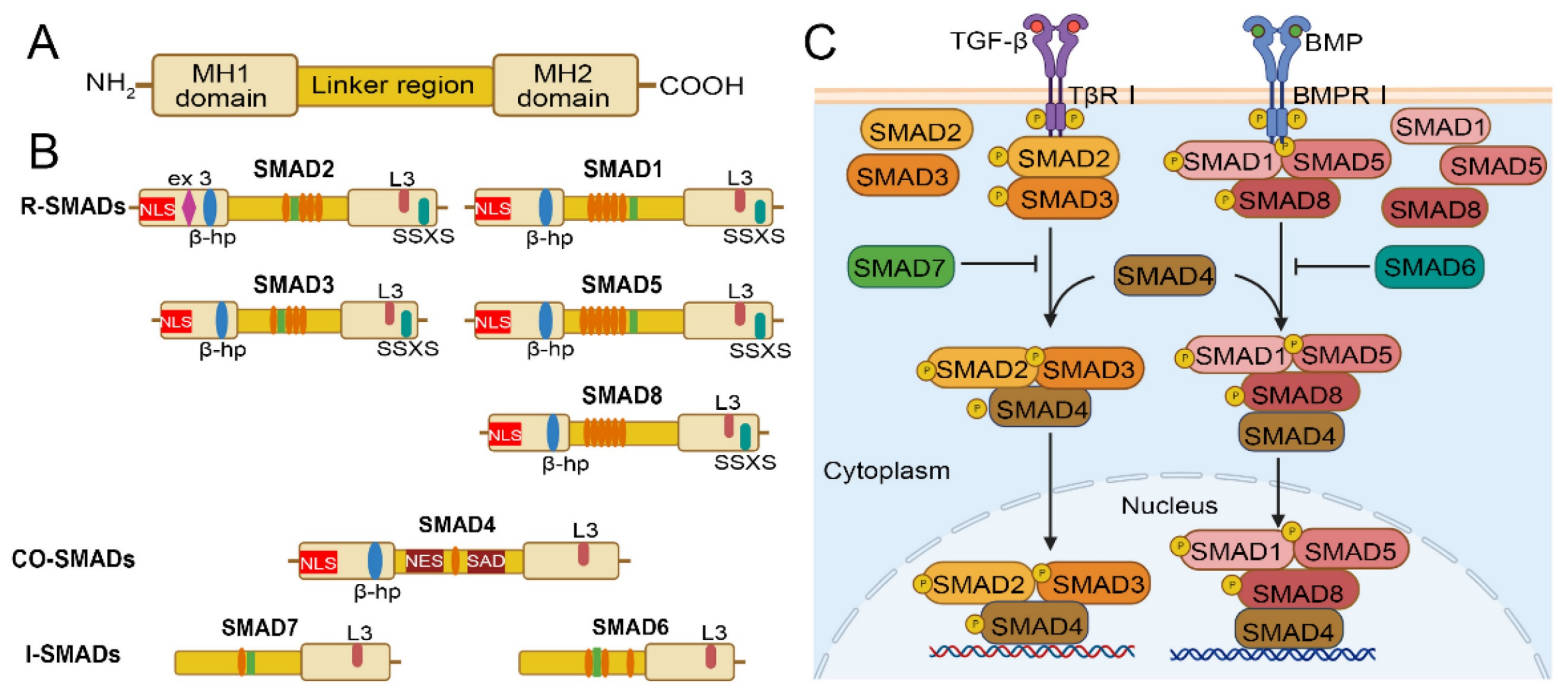
Structure of the SMAD family and overview of TGF-β/SMAD and BMP/SMAD signaling pathway. (A) Structure of the SMAD family. (B) Comparison of the structures of R-SMAD, co-SMAD, and I-SMAD. Elliptical circles in the linker region indicate the PXS/TP (or S/TP) motif potentially phosphorylated by MAP kinases, and the square indicates the PY motif. (C) The TGF-β signaling pathway initiates with the binding of the TGF-β ligand to TβRI. Upon phosphorylation by TβRI, R-SMADs, SMAD2/3, (SMAD1/5/8 phosphorylation by BMP pathway) form a transcriptional complex with the co-SMAD, SMAD4. This complex then translocates into the nucleus, associates with DNA by cooperating with various cofactors, and regulates target gene expression. SMAD6 and SMAD7, the I-Smads, function as negative regulators of TGF-β/SMAD or BMP/SMAD signaling.

SMADs proteins are composed of two spherical structures and an intermediate linking region, the spherical structure includes the amino terminal Mad homology (MH1) domain and the carboxyl terminal MH2 domain^31^ (Figure 1A). MH2 domain is found in all three SMAD types, whereas MH1 domain is preserved exclusively in R-SMADs and co-SMADs. In contrast, the N-terminal regions of I-SMADs are significantly distinct from those of the other SMAD family members. R-SMADs have a characteristic Ser-Ser-X-Ser sequence (SSXS motif) at their C-terminus, which is phosphorylated by type I receptors. The MH2 domain of SMADs contains an L3 loop that plays an important role in binding to the TGF-β receptor and inducing phosphorylation of R-SMADs^32^. R-SMADs and Co-SMAD have a nuclear localization signal (NLS) and a β-hairpin structure in the MH1 domain. The SMAD activation domain (SAD) of SMAD4 is located at the C-terminal of linker region and is essential for the transcriptional activity of SMAD4^33^. In addition, the key to SMAD signal transduction is the recognition of the SMAD4-MH1 domain by SMAD binding DNA elements (SBE) to complete the binding of SMAD polymers to target DNA^34^. Thus, the MH1 domain plays a dominant role in the entry of R-SMADs and Co-SMADs into the nucleus for binding to target DNA^35^. Moreover, SMAD4 has a nuclear export signal (NES) in the linker region, which allows it to translocate between the cytoplasm and nucleus and differs from SMAD2 and SMAD3^36^. For the I-SMAD, although the MH1 and MH2 domains lack NLS, NES, and SSXS motif, they retain the L3 loop structure that can interact with type I receptors^36^. The linker regions of BMP-specific R-SMADs include PXS/TP (or S/TP) motifs targeted by mitogen-activated protein kinases (MAPK) and glycogen synthase kinase (GSK). The phosphorylation of these motifs promotes the rapid degradation of R-SMADs via the ubiquitin-proteasome pathway. Additionally, a PY motif with the PPXY sequence is identified in all SMADs except for SMAD4 and SMAD8, and it is crucial for mediating interactions with proteins containing WW domains, such as HECT-type ubiquitin ligases^37^ (Figure 1B).

### 2.3 TGF-β/SMAD Signaling Pathway

**Figure 2:**
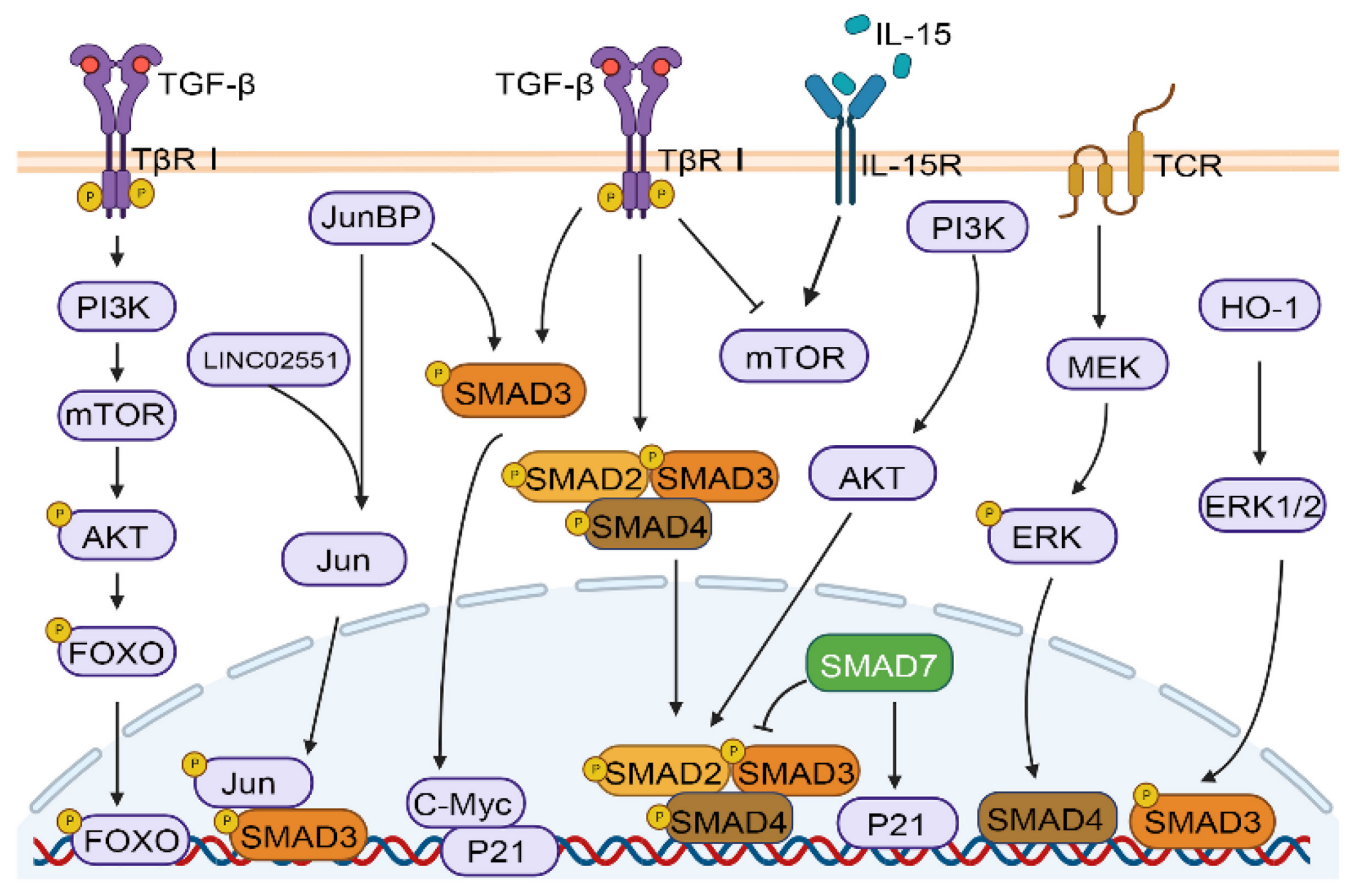
Crosstalk between TGF‑β/SMAD and other pathways. TGF-β/SMAD signaling can crosstalk with other pathways, including the PI3K/AKT, and MAPK pathways (ERK, JNK, and Jun).

The TGF-β signaling pathway plays a crucial role in many physiological and pathological processes, such as embryonic development, tissue homeostasis, immune system, and cancer progression, by activating and transmitting membrane nuclear signals through transcription factors mediated by membrane receptors^10^, ^38^. As shown in Figure 1C, in the classical TGF-β/SMAD signaling pathway, the cytokines of the TGF-β family firstly bind to TβRI and TβRII receptors. TβR I with kinase activity further phosphorylates SMAD2 and SMAD3 proteins, and activated SMAD2 and SMAD3 can combine with SMAD4 to finally complete the formation of SMAD multimers, and then translocate into the nucleus to regulate gene transcription^39^. In addition, the crosstalk between TGF-β/SMAD pathway and other pathways includes but is not limited to MAPK, PI-3 kinase (PI3K)/AKT, and WNT/β-catenin pathways. TGF-β induces Akt phosphorylation through mTOR modulation in a PI3K-dependent manner, resulting in the phosphorylation of forkhead box O (FOXO) transcription factors and consequently regulating the differentiation of induced regulatory T cells (iTregs)^40^. The Jun binding micropeptide (JunBP), encoded by Long intergenic non-protein coding RNA02551 (LINC02551) can enhance the activation of Jun, and further promotes the interaction between SMAD3 and Jun^41^. TGF-β causes the very early inhibition of mammalian target of rapamycin (mTOR) activity in NK cells stimulated with interleukin 15 (IL-15)^42^. Deficiency in retinoic acid-inducible gene I (RIG-I) amplifies TGF-β1-driven phosphorylation of pivotal signaling molecules SMAD2 and Akt in HCC cells^43^. Under the stimulation of heme oxygenase-1 (HO-1), ERK1/2 can phosphorylate SMAD3 at the Thr-179, which may serve as a mechanism for HCC cells to circumvent the growth inhibitory effects of TGF-β1^44^. Furthermore, studies have shown that SMAD4 enucleation in CD8^+^ T cells is regulated by the T-cell receptor (TCR) -MEK/ERK signaling pathway^45^. Furthermore, SMAD3 and SMAD7 are also involved in crosstalk and can facilitate cell cycle arrest through the upregulation of cellular-myelocytomatosis viral oncogene (c-Myc) and P21 expression^46^ (Figure 2).

## 3. The Functional Role of the TGF-β/SMAD Signaling Pathway in HCC

NAFLD and chronic inflammation associated with non-alcoholic steatohepatitis (NASH) are two risk factors for HCC, characterized by hepatocyte steatosis and inflammation, as well as immune cell infiltration^6^. Abnormal events such as chronic liver inflammation, liver fibrosis, and hepatocyte regeneration can lead to cirrhosis. These pathological changes are accompanied by a series of genetic and epigenetic alterations, and eventually result in the early stage of HCC and the formation of 2-3 cm nodules. This genetic change provides a microenvironment for the survival, proliferation, and invasiveness of tumor cells, ultimately leading to irreversible malignant tumor formation^2^, ^47^. The TGF-β/SMAD pathway exhibits different effects on various cells at different stages of inflammation-fibrosis-HCC progression.

### 3.1 The Role of TGF-β in HCC

TGF-β is the core ligand of the TGF-β/SMAD signaling cascade, which is involved in cell proliferation, differentiation, migration, an apoptosis in liver homeostasis, and plays a pleiotropic role in every stage of liver disease progression from steatosis to fibrosis to HCC^48^. The tumor microenvironment of HCC consists of tumor cells, stromal fibroblasts, and infiltrating immune cells. In different circumstances, TGF-β is secreted by multiple cell types in an autocrine and paracrine manner^10^.

**Figure 3:**
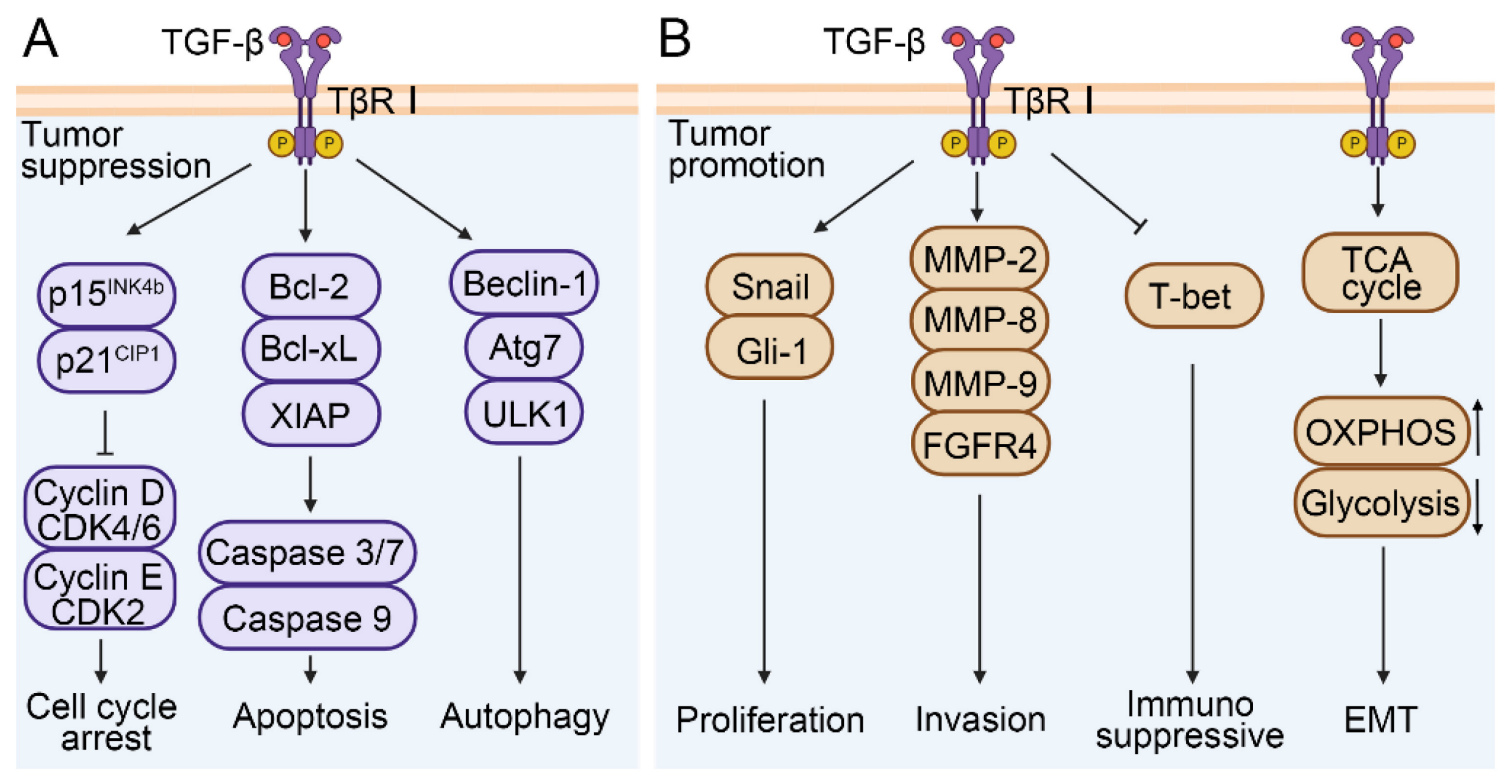
A simplified view of the dual regulatory role of TGF-β in promoting and inhibiting HCC. (A) TGF-β signaling exerts anti-tumor effects by promoting cell cycle arrest, apoptosis, and autophagy. (B) The promoting effects of TGF-β signaling are manifested in the facilitation of cell proliferation, invasion, EMT and immunosuppression in HCC.

TGF-β signaling plays a dual role in the development of HCC. In normal or precancerous hepatocytes, TGF-β signaling causes cell cycle arrest and exerts an anti-tumor effect^49^. TGF-β can inhibit the activity of cyclin D-cyclin dependent kinase 4/6 (CDK4/6) and cyclin E-cyclin dependent kinase 2 (CDK2) complexes by increasing p15^INK4b^ and p21^CIP1^ levels respectively, thereby inhibiting the release of E2 factor (E2F) transcription factors and inducing cell cycle arrest^50^-^52^. TGF-β can reduce the expression levels of B cell lymphoma-2 (Bcl-2), B-cell lymphoma-xL (Bcl-xL), and X-linked inhibitor of apoptosis protein (XIAP) in both normal and malignant hepatocytes, and interfere with the activity of caspase 3/7 and caspase 9, thereby promoting apoptosis^53^. In addition, TGF-β enhances the expression of autophagy-related genes, including Beclin-1, autophagy-related 7 (Atg7), UNC-51-like kinase 1 (ULK1) in HCC cells^53^ (Figure 3A).

During the developmental and metastatic stages of HCC, TGF-β signaling exhibits a role in promoting tumor cell proliferation, EMT, and invasion (Figure 3B). TGF-β was able to activate the expression of zinc finger transcription factor (Snail) and glioma-associated oncogene homolog-1(Gli-1) and induce the proliferation of hepatoma cells, and further promote the development process of HCC^54^, ^55^. TGF-β was also involved in the reprogramming of the tumor microenvironment, such as activating stromal cells, promoting immune escape, increasing ECM deposition, and inducing angiogenesis. For instance, in a CCl_4_-induced mouse model of liver fibrosis, TGF-β1 promoted the differentiation of hepatic interstitial cells into hepatic stellate cells (HSCs) and myofibroblasts^56^. The degradation of ECM by matrix metalloproteinases (MMPs) is one of the key steps of tumor cell invasion and metastasis. Studies indicate that TGF-β can stimulate the expression of MMP-2, MMP-8, and MMP-9 in HCC, and promote the migration and invasion of HCC cells via ERK pathway-mediated fibroblast growth factor receptor 4 (FGFR4) expression^57^-^59^. It was reported that various malignant tumor was associated with increased secretion and activity of MMPs^60^. Additionally, TGF-β also plays an immunosuppressive role in the tumor microenvironment. It can downregulate the expression of T-box expression in T cells (T-bet) and promote the differentiation from Th1 to Th2 cells^29^, ^61^. Similarly, the synergism between TGF-β and IL-21 can promote the differentiation of naïve CD4^+^ T cells into Th17 cells, which contributes to the development of NAFLD-related liver inflammation and HCC^62^. It has also been reported that TGF-β induces naïve B cells to differentiate into immunoglobulin A (IgA) cells during B cell maturation, which is associated with NAFLD-related HCC^63^. Zhang et al. showed that the gene HBx encoded by HBV was involved in angiogenesis and immune escape of the HCC tumor microenvironment through the IL8/CXCR1/TGF-β signaling axis, thus inducing liver metastasis^64^. Further research showed that in Hep3B cells, TGF-β-induced EMT was involved in HCC progression by influencing cell metabolism, such as activating the tricarboxylic acid cycle (TCA cycle), increasing oxidative phosphorylation (OXPHOS), and reducing glycolysis^65^. Unfortunately, this conclusion has not been verified in vivo. Altogether, these studies indicate that TGF-β is closely related to tumor cell survival, proliferation, EMT, and tumor microenvironment reprogramming, and therefore plays a crucial role in HCC development. It is also expected to serve as a diagnostic or prognostic marker.

### 3.2 The Role of SMADs Proteins in HCC

In this review, we mainly summarize the roles of the TGF-β/SMAD family members SMAD2/3, SMAD4, and SMAD7 in HCC.

#### 3.2.1. The functional role of SMAD2/3 in HCC

**Figure 4:**
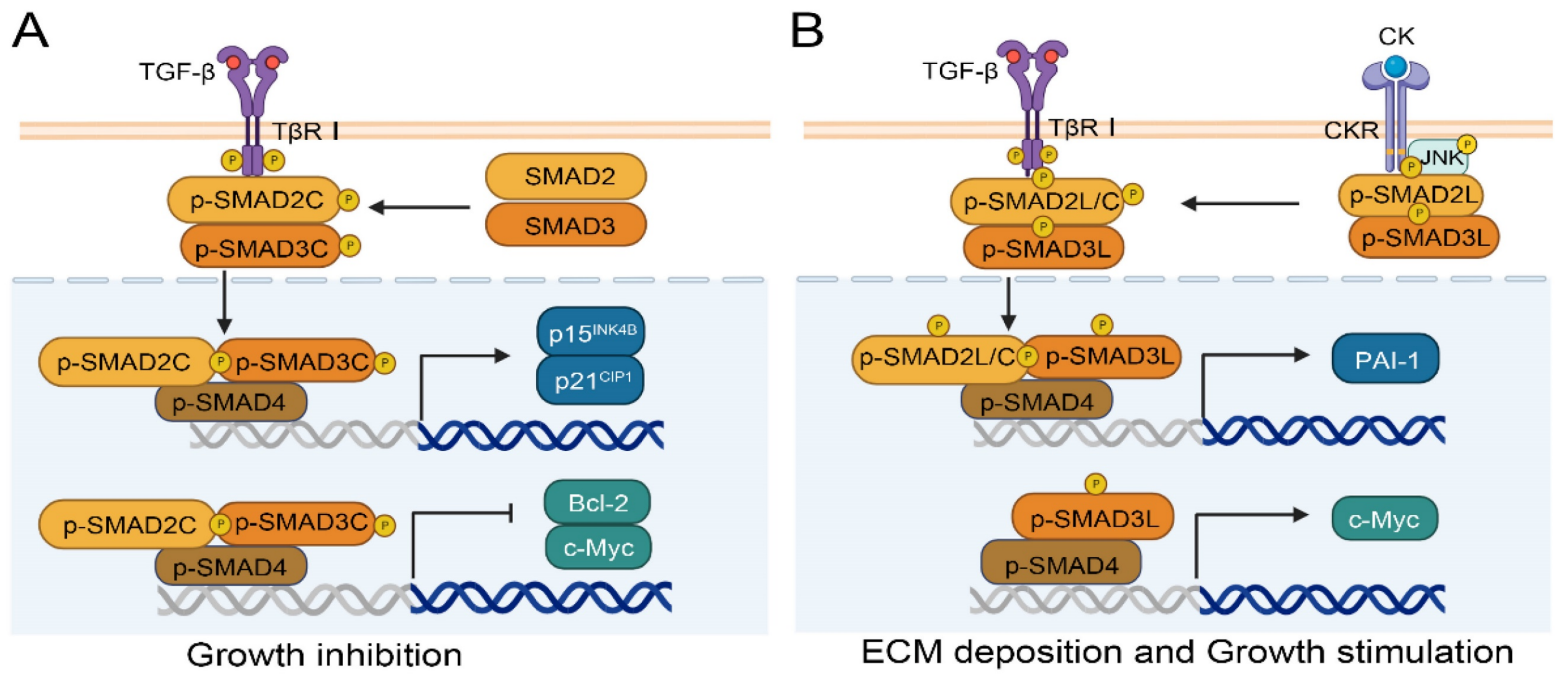
Phosphorylated SMAD2/3 signaling pathways regulate the process of HCC. (A) Phosphorylation at the C-terminal of SMAD2/3 can inhibit the progression of HCC by promoting p15^INK4B^ or p21^CIP1^ and inhibiting Bcl-2 or c-Myc. (B) Phosphorylation of SMAD2/3 in the Linker region facilitates HCC by promoting PAI-1 or c-Myc.

SMAD2/3 are two important downstream genes of the TGF-β signaling pathway that are associated with liver fibrosis and carcinogenesis^66^. The phosphorylation of SMAD2/3 different domains results in different effects in normal and cancer cells. There are three types of phosphorylation isoforms, C-terminal phosphorylated SMAD2/3 (pSMAD2C and pSMAD3C), linker region phosphorylated SMAD2/3 (pSMAD2L and pSMAD3L), and bisphosphorylated SMAD2/3 (pSMAD2L/C and pSMAD3L/C)^67^. The pSMAD3C plays a crucial role in the protection against liver injury and tumor development. Ding et al. confirmed the protective effect of pSMAD3C in liver injury using SMAD3 HT mice with Ser422/423/425 mutations at the C-terminal phosphorylation site of the SMAD3 gene. Their results showed that serum transaminase, inflammatory factors interleukin 6 (IL-6) and tumor necrosis factor alpha (TNF-α) increased in SMAD3 HT mice with C-terminal mutation^68^. Likewise, the TGF-β dependent pSMAD3C signaling pathway promotes apoptosis by regulating apoptosis related genes, including activating p15^INK4B^ and p21^CIP1^^69^, ^70^ or inhibiting c-Myc^71^ and Bcl-2^72^ (Figure 4A). Compared to pSMAD3C, pSMAD3L and pSMAD2L/C have higher carcinogenic potential^73^, and are highly expressed in human HCC cell lines HepG2, Hep3B, and HuH7^74^. Importantly, patients with pSMAD3C have better clinical prognoses than those with pSMAD3L and pSMAD2L/C^75^. As shown in Figure 4B, pro-inflammatory cytokines (CK), such as TNF-α from hepatocytes and myofibroblast, can activate c-Jun N-terminal kinase (JNK) and simultaneously phosphorylate the linker region of SMAD2/3, and ultimately translocating into the nucleus. SMAD complexes stimulate the transcription of plasminogen activator inhibitor type-1 (PAI-1) and the synthesis of ECM, and further promote fibrosis-associated liver cancer^73^. Moreover, the p-SMAD3L-SMAD4 complex can upregulate the transcription of the c-Myc gene and promote cell proliferation, meanwhile inhibiting pSMAD3C-induced apoptosis^73^.

Numerous studies have shown that abnormal activation of SMAD2/3 usually promotes the progression of liver fibrosis. Chemical toxins, such as ochratoxin (OTA), perfluorooctanesulfonic acid, and endosulfan, can activate the TGF-β/SMAD signaling pathway by phosphorylating SMAD2/3, subsequently leading to massive accumulation of ECM, which further aggravates CCl_4_-induced hepatic fibrosis^76^-^78^. Clusterin (CLU) is a secreted protein that can prevent steatohepatitis when overexpressed in hepatocytes. Seo et al. reported that CLU attenuated thioacetamide-induced liver fibrosis in mice by downregulating SMAD3 phosphorylation and its nuclear translocation^79^. Specifically, it is suggested that the activation of SMAD2/3 plays a role in liver fibrosis. At the same time, many studies also support the tumor-promoting effects of SMAD2/3 in HCC. Shu et al. found that selenium phosphate synthetase I (SEPHS1) regulated the expression of SMAD2, SMAD3 and SMAD4, and enhanced the migration and invasion potential of HepG2 cells 80. Dai et al. demonstrated that the overexpression of a DNA and RNA binding protein (KIN17) in liver cancer tissues stimulated the TGF-β/SMAD2 pathway in MHCC-97L and HepG2 cells, and participated in EMT, and ultimately promoted the migration and invasion of HCC cells^81^. Huang et al. found that miR-1258 could directly bind to SMAD2/3 and then counteract the migration inhibition of Hep3B and SMMC-7721 by microRNA-1258^82^. On the other hand, SMAD2/3 had tumor-suppressive effects in HCC. When investigating the effect of Propofol on the proliferation and apoptosis of HCC cells, Li et al. found that Propofol could increase the activity and expression level of TGF-β1 by 12% and 20% respectively and inhibit the proliferation of HCC cells by 10% through the TGF-β1/SMAD2 signaling pathway^83^.

#### 3.2.2. The functional role of SMAD4 in HCC

SMAD4, a DNA-binding protein, forms a multimer with other SMAD proteins such as SMAD2/3 to translocate into the nucleus and transcriptionally regulate downstream target genes^34^. As an important general regulator of the TGF-β/SMAD signaling pathway, SMAD4 plays a complex role in the development of HCC^84^. It can regulate the transcription of many genes positively or negatively, thereby exhibiting pleiotropic effects in HCC.

Specifically in HCC patients, the increased expression of SMAD4 and p-SMAD2/3 is significantly involved in poor postoperative prognosis. It has also been proven that the knockdown of SMAD4 in human HCC cell lines Huh7 and Huh6 reduces colony formation and migration^85^. Yuan et al. found that the survival rate of HCC patients with high expression of SMAD4 and TGF-β was significantly lower than that of patients with low expression by analysing The Cancer Genome Atlas (TCGA) database^86^. Moussa et al. reported that the expression of SMAD4 was significantly higher in advanced fibrosis than that in early fibrosis in chronic hepatitis C lesions^87^. In summary, the above studies indicate that SMAD4 is upregulated in human HCC tissue, which is associated with poor tumor differentiation and prognosis. Patients with high SMAD4 expression have a lower postoperative survival rate than those with low SMAD4 expression.

Moreover, the functional role of SMAD4 in the development of liver diseases has also been investigated by SMAD4 knockout mouse model in our previous work. We constructed a mouse model with the hepatocyte-specific knockout of SMAD4 gene (SMAD4^Δhep^) and induced liver fibrosis using CCL_4_, and found that the expression of SMAD4 was significantly enhanced in the fibrotic liver compared to normal liver tissue. Compared with wild-type mice (SMAD4^fl/fl^), the expression of fibrosis-related markers such as α-smooth muscle actin (α-SMA), type I collagen (Col-I), as well as inflammatory cell markers such as F4/80^+^, CD11b^+^, and Gr1^+^ was significantly increased in SMAD4^Δhep^ mice^88^. In line with this finding, Qin et al. reported that SMAD4 deletion in hepatocytes inhibited adipogenesis, stimulated β-oxidation, improved lipid metabolism, alleviated inflammation and fibrosis, and reduced apoptosis in NASH models constructed by a high-fat diet (HFD) or a methionine and choline-deficient diet (MCD)^89^. These results suggest that SMAD4 promotes liver fibrosis and inflammation in mice, thereby increasing the risk of HCC.

**Figure 5:**
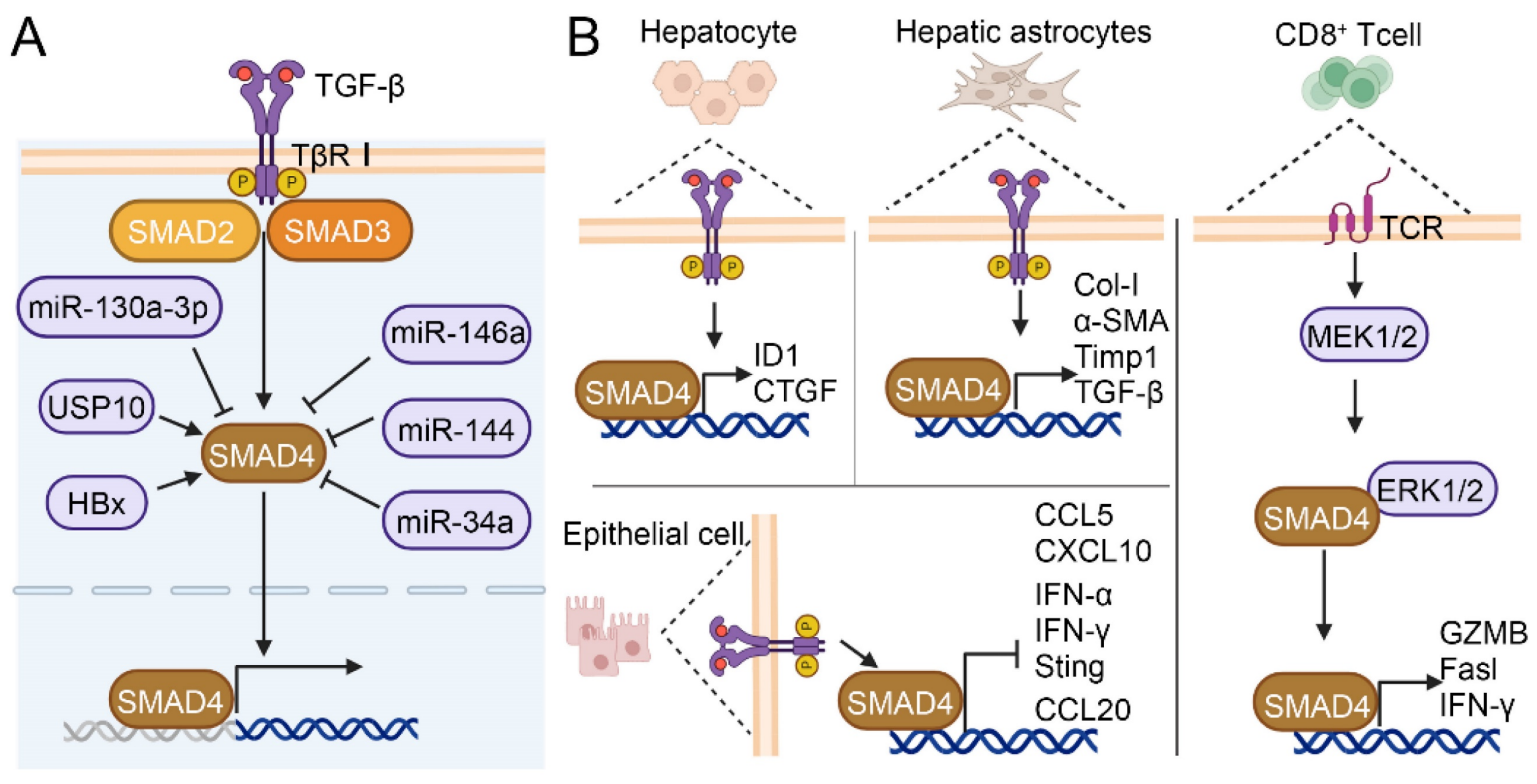
The regulatory role of SMAD4 in HCC and its regulatory effects in different cell types. (A) The interactions between SMAD4 and other proteins, including microRNAs, USP10, and HBX in HCC. (B) The functional role of SMAD4 in hepatocyte, hepatic astrocytes, CD8^+^ T cell and epithelial cell.

SMAD4 can interact with other proteins or molecules in HCC. As shown in Figure 5A, ubiquitin-specific protease 10 (USP10), which is abnormally activated in a variety of malignant tumor, can bind specifically to SMAD4 and stabilize the effect of SMAD4 by increasing its deubiquitination, and further lead to the metastasis of human hepatoma cells Bel-7402^86^. In HBV-associated HCC, the HBV-encoded X protein (HBx) induces transcription and deubiquitination of SMAD4, thereby promoting the replication of HBV^90^. In human gemcitabine-resistant HCC cell lines HepG2 and SMMC-7721, SMAD4 facilitates migration, invasion and EMT. However, microRNA-130a-3p can reverse gemcitabine-resistant by selectively downregulating the expression of SMAD4^91^. MicroRNA-146a and microRNA-144 can also directly target the expression of SMAD4 to reduce liver fibrosis, and play a tumor-suppressive role in the development of HCC^92^, ^93^. Zhang et al. found that long non-coding RNA (LncRNA34a) inhibited the expression of microRNA-34a through promoter methylation and histone deacetylation, thereby weakening the inhibitory effect of microRNA-34a on the target gene SMAD4 and boosting the expression of SMAD4 downstream genes that were related to bone metastasis, ultimately promoting bone metastasis in HCC^94^. The above findings suggest that SMAD4-targeted regulation may be an effective strategy to inhibit HCC progression and metastasis.

SMAD4 plays different roles in different cell types (Figure 5B). The specific knockout of SMAD4 in hepatocytes attenuated CCl_4_-induced liver fibrosis through upregulating the expression of inhibitor of differentiation 1 (ID1) and connective tissue growth factor (CTGF) in hepatocyte^88^. Liu et al. reported that TCR-triggered MEK/ERK signaling in CD8^+^ T cell was able to phosphorylate SMAD4 Ser367 and upregulate TCR signaling pathway members such as cluster of differentiation 247 (Cd247), phosphoinositide-3-kinase regulatory subunit 1 (Pik3r1), salt overly sensitive 2 (Sos2), and cytotoxicity-related genes such as Granzyme B (GZMB), factor related ligand (Fasl) and interferon-γ (IFN-γ), thus enhancing CD8^+^ T cell effect function^45^. Khanizadeh et al. found that SMAD4 deletion in human HSCs cell line LX-2 downregulated the mRNA levels of fibrosis-related genes Col-I, α-SMA, TGF-β, and Timp1^95^. Moreover, research indicated that SMAD deficiency in pancreatic ductal adenocarcinoma (PDAC) cells activated IFN-I and stimulator of interferon genes (STING) signaling to increase the expression of C-C motif chemokine ligand 5 (CCL5) and C-X-C motif chemokine ligand 10 (CXCL10), thereby enhancing immune recognition and improving the antigenicity of tumor cells^96^. SMAD4 silence in colon epithelial cell promoted the expression of inflammatory gene C-C motif chemokine ligand (CCL20), which led to susceptibility to colitis-associated cancer^97^. Although the SMAD4-mediated TGF-β signaling pathway is well elucidated, it is not yet clear how SMAD4 contributes to different disease stages in different cells and pathways. In conclusion, it is crucial to conduct in-depth research on the functional role of SMAD4 in different cells during the development of HCC.

#### 3.2.3. The functional role of SMAD7 in HCC

SMAD7 is a negatively regulated component in liver fibrosis and HCC. A Kaplan-Meier analysis of 140 HCC patients demonstrated that low expression of SMAD7 was closely related to reduced survival^98^. Furthermore, it has also been confirmed that SMAD7 deficiency enhances cell proliferation by activating TGF-β and nuclear factor-κB (NF-κB) signaling pathways in N-nitrosodiethylamine (DEN)-induced HCC mouse model with systematic SMAD7 knockdown^99^. Meanwhile, in vitro experimental results also supported that overexpression of SMAD7 led to decreased expression of Col I, α-SMA, and hydroxyproline^100^.

SMAD7 binds to different synergistic effectors in different cells and involves different gene expressions and different cellular processes. Interestingly, SMAD7 deficiency in hepatocytes accelerated DEN-induced HCC development by activating the STAT3 signaling pathway^46^. This result further supports the conclusion that SMAD7 has tumor inhibitory function. In contrast, macrophage-specific SMAD7 deletion did not affect alanine aminotransferase (ALT) and aspartate transferase (AST) levels, inflammatory cell infiltration, and fibrosis degree in CCL_4_-induced chronic liver injury mouse models^101^. In addition, the evidence also demonstrates that SMAD7 can bind to the self-renewal genes Nanog, sex determining region Y-box 2 (Sox2), and inhibitor of DNA binding 2 (ID2) in embryonic stem cells (ESCs), regulating the self-renewal and pluripotency of ESCs^102^.

### 3.3 Inter-Regulation between TGF-β/SMAD Signaling Pathway and MicroRNAs in HCC

MicroRNAs are non-coding single-stranded RNA molecules with 19 to 25 nucleotides in length. They can bind to mRNA to interfere with translation, and thereby regulate gene expression. In a comprehensive analysis of genomic and epigenomic data from 377 HCC patients, Yang et al. identified 88 microRNAs as driving factors for HCC^103^. Furthermore, evidence suggests that microRNAs play a regulatory role not only in TGF- β/SMAD signaling pathway but also in other signaling pathways, including cell cycle, Wnt signaling, and janus kinase (JAK)-signal transducer and activator of transcription (STAT) signaling^103^. Thus, microRNA dysregulation and crosstalk with other signaling pathways are closely correlated with the progression of HCC. Research indicates that the expression of microRNAs plays a dual role in promoting or inhibiting HCC^104^.

**Figure 6:**
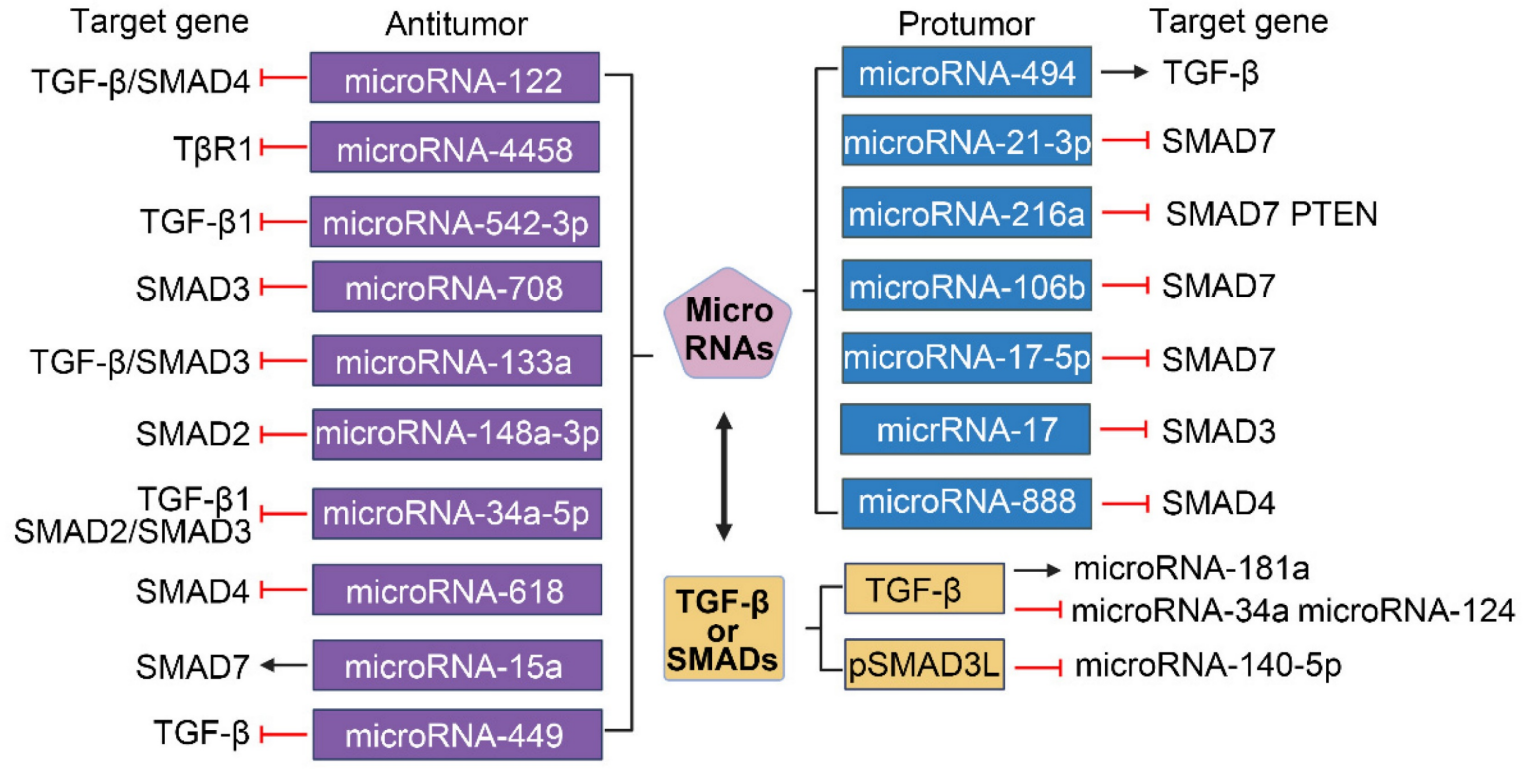
Crosstalk between TGF-β/SMAD and MicroRNAs. Numerous microRNAs have been implicated in the regulation of HCC. MicroRNAs are categorized based on their influence on tumorigenesis: those with tumor-suppressive roles are highlighted in purple, while those with tumor-promoting effects are denoted in blue. The transcription of microRNAs (indicated in yellow) is modulated by the TGF-β/SMAD signaling pathway, which can exert either upregulatory or downregulatory effects.

The promoters of several microRNAs that target the TGF-β/SMAD pathway play different roles in HCC^105^. As shown in Figure 6, miroRNA-122, microRNA-4458 and microRNA-542-3p inhibit the migration, invasion and EMT of HCC cells by suppressing the TGF-β signaling pathway via targeting TGF-β or TβR1^106^-^109^. MicroRNA-708 expression leads to decreased proliferation, migration, and invasion of HCC cells through directly targeting SMAD3^110^. Overexpression of microRNA-133a suppresses cell proliferation, migration, invasion and induces cell apoptosis by targeting Fos-related antigen 2 (FOSL2) through TGF-β/Smad3 signaling pathway^111^. MicroRNA-148a-3p represses the development of HCC by targeting the expression of SMAD2^112^. MicroRNA-34a-5p and microRNA-618 can downregulate the expression of Col I and α-SMA in HSCs and alleviate liver fibrosis by targeting SMAD4 in the TGF-β/SMAD signaling pathway, which suggests that the interaction between microRNA and TGF-β/SMAD also plays a role in HSCs activation^113^, ^114^. Additionally, ex vivo and in vivo experiments have revealed that the expression of SMAD7 in HBV-related HCC tissue is positively correlated with microRNA-15a^115^. MicroRNA-449a, microRNA-449b, and microRNA-449c can directly target SOX4 and indirectly interfere with the pro-HCC pathway of TGF-β by inhibiting TGF-β-mediated SOX4 overexpression and cell migration^116^. MicroRNA-494 can upregulate the downstream gene TGF-β by binding to sirtuin 3 (SIRT3), and then promote the development of HCC by inducing EMT^117^. Hong et al. showed that microRNA-21-3p downregulated SMAD7 to relieve the inhibitory effect of SMAD7 on yes-associated protein 1 (YAP1), thereby exerting carcinogenic effects^118^. Upregulation of phosphatase, tensin homolog (PTEN) and SMAD7 by microRNA-216a/217 contributes to enhance drug resistance and recurrence in HCC^119^. Li et al. demonstrated that SMAD7 was predicted as a target of microRNA-106b and could reverse the promotive effects of microRNA-106b on HCC cell progression and EMT^120^. In addition, the expression of microRNA-17-5p is strongly upregulated in liver fibrosis, and TGF-β1-induced expression of Col-1 and α-SMA in HSCs is facilitated by microRNA-17-5p-mediated downregulation of SMAD7^121^. MicroRNA-17 and microRNA-888 promote cell migration and invasion of HCC by directly targeting SMAD3 and SMAD4^122^, ^123^.

TGF-β/SMAD signaling can also act as an effector of microRNA in HCC events (Figure 6). In HSCs, experimental downregulation of augmenter of liver regeneration (ALR) can naturally occur in the process of liver fibrosis, and promote the expressions of Col I, α-SMA, and ras-related C3 botulinum toxin substrate 1 (rac1), which is correlated with microRNA-181a induced by TGF-β^124^. Hu et al. indicated that pSMAD3L was able to activate the EMT process by downregulating microRNA-140-5p expression in a DEN-induced rat model^125^. Furthermore, the crosstalk between TGF-β/SMAD signaling and microRNA also exists in the tumor microenvironment. Such as TGF-β leads to increased production of chemokine C-C motif chemokine ligand 22 (CCL22) by downregulating the expression of microRNA-34a in HBV-associated HCC, thereby recruiting regulatory T cells (Tregs) into the tumor microenvironment to promote immune escape^126^. TGF-β1 can induce the MAPK pathway to promote tumorigenesis and progression in HCC by repressing microRNA-124^127^.

## 4. The Role of the TGF-β/SMAD Pathway in the Prevention and Treatment of HCC

Although many factors result in the complexity of the TGF-β/SMAD pathway in the regulation of HCC, the drug research for the HCC treatment by targeting the TGF-β/SMAD pathway shows good prospects. For example, chemotherapy drugs, small molecule inhibitors, therapeutic vaccines, and traditional Chinese medicine preparations exert their effects through specific or non-specific targeting of TGF-β/SMAD (Table 1).

In the management of HCC, specific chemotherapeutic agents have demonstrated efficacy in halting tumor proliferation and metastasis by targeting the TGF-β/SMAD signaling cascade. Fluorofenidone (AKF-PD), an established drugs for idiopathic pulmonary fibrosis, also offers potential benefits for liver fibrosis by repressing HSC autophagy through the attenuation of the TGF-β1/SMAD pathway, potentially impeding the progression to HCC^128^. Sanguinarine (San), a natural benzo phenanthridine alkaloid, restrains tumor growth and HIF-1α signaling, and suppresses the expression changes of EMT markers as well as blocks p-SMAD2/3 translocation to the nucleus. It can also inhibit TGF-β-induced cell migration in HCC cells^129^. According to current research, aspirin can alleviate liver fibrosis induced by thioacetamide (TAA) by reducing the secretion of TGF-β1 in activated HSCs and Kupffer cells. It also downregulates the expression of p-SMAD2, p-SMAD3, α-SMA, and Col I in rats^130^. Praziquantel (PZQ) is a widely used antiparasitic drug that has been found to have additional beneficial effects, including the amelioration of liver fibrosis. PZQ can effectively inhibit the TGF-β/SMAD signaling pathway, thereby leading to a decrease in HSCs activation and collagen production by upregulating SMAD7^131^. Ursodeoxycholic acid (UDCA) is a secondary bile acid derived from ursodeoxycholic acid, which is converted by intestinal bacteria. Studies have found that UDCA enhances anti-tumor immunity by inhibiting the differentiation and activation of Tregs in a TGF-β-dependent manner^132^. Currently, there are no immune therapy drugs targeting the TGF-β/SMAD signaling pathway directly for the treatment of HCC. However, there are some immune therapy approaches under investigation that may indirectly affect the TGF-β/SMAD signaling pathway. Immune checkpoint inhibitors, such as anti- programmed death 1 (PD-1) and anti-PD-L1 antibodies, are a commonly used class of immune therapy drugs. Some studies have shown that a novel anti-TGF-β/vascular endothelial growth factors (VEGF) bispecific antibody Y332D combined with PD-1 blockade exerts superior antitumor effect through improving immune microenvironment^133^.

Targeting the TGF-β/SMAD signaling pathway with small molecule inhibitors is an emerging therapeutic strategy for HCC. These drugs inhibit key molecules in the TGF-β signaling pathway, reducing the proliferation and metastatic ability of tumor cells, thus inhibiting the development of HCC. Galunisertib (LY2157299) is a small molecule inhibitor of the TGF-β signaling pathway that acts by downregulating TβRI and is used to treat advanced HCC patients^134^. Similarly, Galunisertib can also inhibit the HCC aggressiveness by modulating CD44 expression and reducing stemness-related functions^135^. Moreover, it was proved that Galuniserib could effectively enhance the anti-tumor activity of HCC patients in stereotactic radiotherapy (SBRT)^136^. Galuniserib also reduced disease progression by downregulating EMC deposition in a liver injury mouse model with multidrug resistance 2 (Mdr2) deficient^137^, ^138^. LY2109761, an orally active TβRI/II kinase dual inhibitor, had inhibitory effects on HCC. It has been reported that it can suppress cell migration by specifically inhibiting SMAD2, one of the downstream effectors of TGF-β signaling^139^. In addition, SKLB023 is a novel small molecule inhibitor of inducible nitric oxide synthase (iNOS) that can block joint inflammation and cartilage damage in arthritis. Importantly, studies have indicated that SKLB023 has significant effects in liver fibrosis treatment by inhibiting the expression of TGF-β and decreasing the phosphorylation levels SMAD2/3^140^. Interestingly, LY3200882, a novel next-generation TβRⅠ small molecule inhibitor, is currently undergoing clinical trials for the treatment of various tumors^141^.

Therapeutic vaccines targeting the TGF-β/SMAD signaling pathway for HCC are a forefront treatment strategy. Belagenpumatucel-L (Lucanix) is a homozygous tumor vaccine prepared from four irradiated human non-small-cell carcinoma (NSCLC) cell lines (SK-LU-1, NCI-H460, NCI-H520, Rh2) that have been modified with TGF-β2 antisense plasmid^142^. Notably, Lucanix enhances the immunogenicity of allogeneic lung cancer vaccine cells by blocking the expression of TGF-β, thereby generating an anti-tumor immune response^142^.

TCM exert inhibitory effects by targeting the TGF-β/SMAD signaling in HCC and other liver diseases. For instance, the Herbal formulation compound kushen injection (CKI) inhibits HSCs activation by stabilizing SMAD7 and downregulating SMAD2/3 phosphorylation^143^. Zheng et al. showed that Songyou Yin could significantly downregulated TGF-β-induced EMT, invasion, and migration in MHCC97H cells after 4 weeks of treatment^144^. Echinacoside (ECH) is the main active ingredient of Cistanche. It can reduce the transcription of genes involved in cell proliferation, migration, and metastasis by microRNA-503-3p/TGF-β1/SMAD axis, and provide a safe and effective anti-tumor drug active ingredient for HCC^145^. Similarly, Tanshinone IIA (Tan IIA) can also repress the proliferation, migration, and invasion of HCC and promote apoptosis by regulating the expression of SMAD7 and YAP in a TGF-β signaling-dependent manner^146^. Hesperidin, a plant-derived bioflavonoid with anti-tumor properties, can activate nuclear factor E2-related factor 2 (Nrf2) and peroxisome proliferator-activated receptor γ (PPARγ), and downregulate TGF-β1/SMAD3 signaling, which is correlated with the reduction of oxidative stress and the inactivation of pro-survival mechanisms in the liver^147^. Shikonin (SHK) has been found to effectively suppress the progression and EMT of HCC through the modulation of the miroRNA-106b/SMAD7/TGF-β pathway^120^. Interestingly, some TCM act therapeutically by promoting pSMAD3C and inhibiting pSMAD3L. For instance, Salvianolic acid B (Sal B), a major phenolic compound, is accepted as an effective drug for hepatic disorders. Research indicates that Sal B enhances the conversion of SMAD3 phosphorylation from the oncogenic JNK/pSMAD3L/c-Myc pathway to the tumor-suppressive TβRI/pSMAD3C/p21 pathway, highlighting the pivotal role of pSmad3C's in therapy^148^, ^149^. Compound astragalus and salvia miltiorrhiza extract (CASE) has been shown to inhibit the migration, proliferation, and growth of HepG2 cells stimulated by TGF-β1. This effect is achieved by increasing the levels of microRNA-145, which regulates the signal switch from pSMAD3L to pSMAD3. Additionally, CASE decreases the levels of microRNA-21 and inhibits the phosphorylation of SMAD3L that is dependent on MAPK^150^. Similarly, Astragaloside IV (AS-IV), a major compound extracted from astragalus membranaceus, has demonstrated promising anti-cancer effects in various types of cancer. Recent studies have confirmed that AS-IV can suppress the migration and invasion of HuH-7 cell by the upregulation of the pSMAD3C/p21 pathway and the downregulation of the pSMAD3L/c-Myc pathway^151^. These treatments targeting the TGF-β/SMAD pathway highlight the synergistic effects in HCC therapy.

## 5. Discussion

The increasing incidence and mortality of liver cancer remain a major global challenge^1^, ^3^. Due to the complexity of the pathogenic factors and mechanisms, humans face many difficulties in combating liver cancer. The role and mechanism of TGF-β/SMAD pathway members under physiological and pathological conditions have become a hot research topic. Undoubtedly, many factors, such as TGF-β activity, TGF-β receptor stability, SMAD transcription activity, and upstream or downstream regulatory factors that affect SMAD protein transcription, determine the pleiotropy and complexity of TGF-β/SMAD signaling in HCC. This review focuses on the functional role, mechanism, and potential application of the TGF-β/SMAD signaling in HCC. In general, the TGF-β/SMAD signaling plays a pivotal role in accelerating proliferation, invasion, and metastasis by promoting angiogenesis, suppressing anti-tumour immunity, and affecting cellular metabolic profiles. Based on in-depth research on the mechanism of the TGF-β/SMAD pathway in HCC, targeted strategies against TGF-β/SMAD have been developed, such as chemotherapy drugs, small molecule inhibitors, therapeutic vaccines, traditional Chinese medicine formulations.

However, there are still many issues to be elucidated about the TGF-β/SMAD signaling in HCC. Firstly, the impact of TGF-β/SMAD on the progression of liver cancer is controversial and even contradictory. Numerous studies have shown that the TGF-β/SMAD signaling pathway is involved in aggressive metastasis and poor survival in HCC^50^, ^85^, but there are also contrary findings^152^. Possible reasons include: (1) the signaling pathway is highly dependent on the environment and disease stage, it exhibits different action phenotypes at different disease stages and in different environment where cells are located; (2) the HCC tumor microenvironment involves the interaction of multiple promoting or carcinogenic signaling pathways, which together determine the progress of the disease. Other signaling members, such as P38/MAPK, JAK/STAT, and PI3K/mTOR, directly influence TGF-β/SMAD signaling through protein-protein interactions or regulating the transcriptional activity of SMAD by downstream transcription factors^153^. Secondly, how members of the TGF-β/SMAD signaling pathway convert from tumor suppression to tumor promotion, and their roles in different cells of the tumor microenvironment need to be further explored. Interestingly, based on preclinical data, inhibition of drug ligands and TGF-β receptors may bring new ideas for liver cancer treatment, and im-prove the efficiency of existing chemotherapy. Therefore, the application of these basic findings to clinical treatment remains a major challenge. Finally, there are still some limitations to targeted therapy against the TGF-β/SMAD signaling pathway. Fresolimumab (GC1008) is a human IgG4 monoclonal antibody that can neutralize all TGF-β subtypes and has shown anti-tumor effects in phase 1 trials in patients with malignant melanoma (MM) and renal cell carcinoma (RCC), but reversible cutaneous keratoacanthoma is a noteworthy side effect^137^. Research has demonstrated that TGF-β signaling, particularly via TβR1, is essential for cardiac development, as evidenced by heart defects in TGF-β2-null mice and cardiovascular side effects, including valve thickening and cellular proliferation, upon treatment with TGF-β blocking antibodies and TβR1 inhibitors like AZ12601011 and AZ12799734^154^. Mouse model experiments also indicated that disrupting TGF-β/SMAD signaling, such as the absence of SMAD4 in T cells, could cause severe gastrointestinal inflammation and precancerous lesions in the gastroduodenal area^155^. Although the development of drugs targeting the TGF-β pathway is complex and risky, the development of strategies to block TGF-β signaling and combination therapy is still progressing in clinical trials. This is because there is a growing recognition that targeting the TGF-β/SMAD signaling pathway has significant implications in cancer treatment. By inhibiting this pathway, tumor infiltration can be reduced and treatment effectiveness can be improved, especially in solid tumors. This approach holds great promise for improving cancer treatment outcomes. In conclusion, targeting TGF-β/SMAD signaling is a new opportunity for personalized medicine and the development of novel therapeutic interventions.

## Figures and Tables

**Table 1:** Summary of chemotherapy drugs, small molecule inhibitors, therapeutic vaccines, and traditional Chinese medicine formulas that exert anti-tumour effects through the TGF-β/SMAD signaling pathway.

Name	Targets	Mechanism of action	References
Chemotherapy drugs			
Fluorofenidone	TGF-β1/SMAD	Inhibition of HSCs autophagy	128
Sanguinarine	p-SMAD2/3	Reduce the expression of EMT-related genes Inhibition of cell migration	129
Aspirin	TGF-β1, p-SMAD2/3	Inhibition of HSCs activation	130
Praziquantel	SMAD7	Inhibition of HSCs activation	131
Ursodeoxycholic acid	TGF-β	Inhibition of differentiation and activation of Tregs	132
Small molecule inhibitors			
Galunisertib	TβRI	ECM deposition	135, 137, 138
LY2109761	TβRI/II, SMAD2	Inhibition of cell migration	139
SKLB023	p-SMAD2/3	Inhibition of HSCs activation	140
LY3200882	TβRI	Reduce DNA damage and increase cell death	141
Therapeutic vaccines			
Belagenpumatucel-L	TGF-β2	Block TGF-β expression	142
Traditional Chinese Medicine			
Compound kushen injection	SMAD7, SMAD2 / 3	ECM deposition	143
Songyou Yin	TGF-β1, SMAD2/3	Reduce the expression of EMT-related genes	144
Echinacoside	TGF-β1/SMAD	Inhibition of cell proliferation, invasion, and migration	145
Tanshinone IIA	SMAD7	Inhibition of cell proliferation, invasion, and migration	146
Hesperidin	TGF-β1 / SMAD3	Amelioration of oxidative stress and inflammation	147
Shikonin	SMAD7	Inhibition of EMT	120
Salvianolic acid B	pSmad3C, pSmad3L/c	Inhibition of proliferation and migration	148, 149
Compound Astragalus and Salvia miltiorrhiza extract	pSmad3C, pSmad3L/c	Inhibition of cell migration and proliferation	150
Astragaloside IV	pSmad3C, pSmad3L/c	Inhibition of cell migration and invasion	151
